# A matter of measurement? A Swedish register-based study of migrant residential segregation and all-cause mortality

**DOI:** 10.1016/j.ssmph.2025.101793

**Published:** 2025-03-27

**Authors:** Agneta Cederström, Andrea Dunlavy

**Affiliations:** a Department of Public Health Sciences, Stockholm University, Stockholm, Sweden; b Centre for Health Equity Studies (CHESS), Stockholm University/Karolinska Institutet, Stockholm, Sweden

**Keywords:** Migration, Residential segregation, Measurement, All-cause mortality, Sweden

## Abstract

**Background:**

In recent decades, Sweden has become an increasingly diverse society by origin, but one in which residential segregation by migrant background has also increased. This study examines how different aspects of migrant residential segregation are associated with all-cause mortality among native-born and migrant populations.

**Methods:**

Using Swedish population-based registers, this longitudinal open cohort study assessed associations between four local level indices of migrant residential segregation and all-cause mortality among adult migrant and native-born residents of Sweden's three largest metropolitan areas (Stockholm, Gothenburg, and Malmö) between 2004 and 2016. Multilevel Poisson regression models, adjusted for individual-level sociodemographic and socioeconomic factors as well as area-level socioeconomic conditions, were used to estimate associations between these indices and all-cause mortality.

**Results:**

Moderate decreased mortality risks were observed among migrants in residential areas with higher levels of migrant density, isolation, and exposure in fully adjusted models. However, isolation and exposure effects could not be distinguished due to a high degree of correlation between the isolation and exposure measures. In fully adjusted models mortality gradients were largely unobserved among native-born individuals in relation to migrant residential segregation. The evenness dimension of segregation showed limited relevance for mortality risk in both groups.

**Conclusions:**

This study provides evidence that higher migrant density is associated with lower mortality risks among migrants, suggesting that residential areas with higher proportions of migrants may offer health benefits for migrants. These findings highlight the importance of residential contexts in shaping migrant health outcomes.

## Highlights

• Novel comparisons of multiple measures of residential segregation.
• Associations between residential segregation and mortality differed by nativity.
• Decreased mortality risks seen among migrants in areas of high migrantdensity.
• Negligible associations observed between segregation and mortality in native-born.

## 1 Introduction

In recent decades, the Swedish population has increasingly diversified by country of origin, with over 25% of the current population born in other countries (Statistics Sweden, 2024a). This demographic change has contributed to the formation of an increasingly diverse society by origin, but one that is also characterized by various forms of stratification based on international migrant background, with implications for residential segregation. Migrant residential segregation is prevalent in all of Sweden's largest metropolitan regions, including Stockholm, Gothenburg, and Malmö, where the highest proportions of foreign-born individuals reside (Statistics Sweden, 2024b). Non-European migrants, many of whom are from the Global South (Malmberg et al., 2018; Marcińczak et al., 2015), experience particularly high levels of segregation, and are more likely to reside in peripheral suburbs outside of city centers compared to native-born individuals (Liebig & Spielvogel, 2021). There is also a strong correlation between migrant and income residential segregation in Sweden (Malmberg & Clark, 2021), and neighborhoods with high proportions of residents from non-European countries also often have a high concentration of lower-income earners (Hedman & Andersson, 2016).

Research has shown that residential segregation is an important social determinant of health that can operate through multiple material and psychosocial pathways (Kramer & Hogue, 2009; White & Borrell, 2011; Williams & Collins, 2001). Socioeconomic and ethnic stratification processes can fuel the residential sorting of racial/ethnic minorities and migrants into areas that may also be characterized by disadvantage or deprivation (Charles, 2003; Vogiazides & Chihaya, 2020), such as access to fewer and/or lower quality resources (e.g., health care facilities, education) or greater exposure to environmental and social hazards. Previous studies on ethnic and racial segregation from other country contexts have shown increased risks for a variety of adverse health outcomes among minorities who live in segregated areas, including mortality (Frankenfeld et al., 2022; Poulson et al., 2021), poor self-rated health (Yang et al., 2017) and adverse perinatal health outcomes (Mehra et al., 2017), among others. However, studies of residential composition have suggested protective health effects among ethnic minorities who live in areas with higher proportions of minority or co-ethnic residents (Becares et al., 2012, 2018; Das-Munshi et al., 2012; Schofield et al., 2017, 2024; Seo & Jivraj, 2024), often referred to as the *ethnic density effect*. 10.13039/100001807Protective factors may be related to increased social capital, cohesion, or support from living in close proximity to others with shared ethnic/racial or origin backgrounds (Becares et al., 2012, 2018). These diverging patterns point to the complex relationship between segregation and health, as different aspects of segregation may entail protective or harmful effects for distinct health outcomes (Becares et al., 2012; Kramer & Hogue, 2009; White & Borrell, 2011). Differences across studies in how residential segregation is conceptualized and measured also play a role. Although there is conceptual overlap between residential segregation and composition, residential segregation is a multidimensional construct concerned with the spatial distribution of groups across neighborhoods within a larger area (e.g., metropolitan), whereas residential composition is primarily concerned with the relative size or proportion of groups within a given area (Yang et al., 2020).

The extent to which different dimensions of migrant residential segregation have implications for health has been largely unexplored in Sweden, as studies have focused primarily on residential composition, measured in terms of migrant and (co-)ethnic density. For example, recent findings have shown protective effects of high own-region migrant density on the risk of non-affective psychosis (Dykxhoorn et al., 2020) with similar findings having also been observed internationally (Baker et al., 2021; Schofield et al., 2024). Yet other Swedish studies, focused on specific country of origin groups, have suggested that living in ethnic enclaves (i.e., areas with high co-ethnic density) does not influence the risk of psychiatric disorders (Mezuk et al., 2015) nor the risk of diabetes (Mezuk et al., 2014) among migrant groups; however, these studies found increased risks among the native-born majority populations who lived in these enclaves. This research has contributed much to understanding the links between ethnic density and health, but far less is known about associations with mortality. In addition, the extant literature is largely centered on racial/ethnic segregation from other high-income countries, primarily the United States. Much of this literature focuses on the segregation of racial/ethnic minority groups that comprise considerable proportions of the US population, including Black, Hispanic and Latino, and Asian populations. By contrast, Swedish research focuses on migrant background, and information on self-reported race/ethnicity is not available in Swedish administrative data sources. Sweden's migrant population is heterogenous by origin, reflecting different waves of immigration. Labor migration from Nordic and other European countries was predominant during the mid-twentieth century, but from the late 1970s shifted towards refugee, asylum seeker, and family reunification migration from a variety of countries outside of Europe (Dunlavy, 2017). Immigration peaked in 2016, but has since declined (Statistics Sweden, 2024c). Further, ethnic residential segregation in Sweden is a relatively recent development that is linked to immigration, whereas the US context has a longer history of residential segregation among racial/ethnic minority groups. Levels of urban ethnic residential segregation in Europe have also been characterized as more moderate than those in the United States (Andersson et al., 2018). Thus, the applicability of much of the existing knowledge to the Swedish context is not entirely evident, and points to the need for further investigation.

The aim of this study is to examine relationships between migrant residential segregation and all-cause mortality, a broad indicator of both population health (Parrish, 2010) and health inequalities (Mackenbach et al., 2019). The study is focused on the three largest metropolitan regions in Sweden and extends the segregation and health literature with a more nuanced examination of how distinct forms of migrant residential segregation may be differentially associated with all-cause mortality. As it has previously been suggested that residential segregation may have different impacts on health in minority and majority populations (Kramer & Hogue, 2009), we investigate whether or not nativity and region of origin modify associations between migrant residential segregation and mortality.

## 2 Data and methods

### 2.1 Study population and design

The study utilized administrative data from several longitudinal total population registries that were linked via pseudonymized personal identification numbers. The study population was comprised of adults aged 18 years and above residing in Sweden's largest metropolitan areas (Stockholm, Gothenburg, and Malmö) between 2004 and 2016, the latest year these data were available to us. An open cohort study design was used to allow for the inclusion of persons who immigrated to Sweden or turned 18 during the follow-up period. Person-time was assessed from the year a given person turned 18, follow-up commencement (January 1, 2004), or arrival in Sweden (if 18 years or older and foreign-born) until death, emigration, metropolitan relocation, or end of follow-up (December 31, 2016).

Information on country or region of birth from the Register of the Total Population was used to categorize the study population as native- or foreign-born. The foreign-born population was further distinguished by Global North or Global South origin (United Nations Trade and Development (UNCTAD), 2024). Individuals with missing data on country or region of birth were excluded from the study (N = 313 (0.01%) in Stockholm, N = 54 (0.005%) in Gothenburg, and N = 174 (0.02%) in Malmö).

Ethical approval for the study was granted by the Regional Ethical Review Board in Stockholm, Sweden (decision no. 2017/716–31/5).

### 2.2 Measures of migrant residential segregation

The conceptual complexity of segregation has resulted in the development of multiple measures to assess its many aspects. Seminal work by Massey and Denton (1988) identified five core dimensions of segregation, upon which the majority of traditional segregation indices are based. Yet prior research has posited that these five dimensions can be further condensed into isolation/exposure and evenness (Reardon & O’Sullivan, 2004), and much of the existing evidence has focused on these dimensions. However, as noted above, other segregation studies have specifically assessed the compositional characteristics of residential areas. In the current study, we employ measures which examine three broad aspects of residential segregation, including 1) *composition*, by measuring migrant density; 2) *interaction*, by measuring isolation and exposure, and 3) *distribution*, by measuring evenness. All measures were constructed at the local level, in order to better capture lived experiences of segregation at smaller geographical scales (i.e., neighborhoods) rather than across larger geographic areas (i.e., metropolitan areas).

The local measures of migrant residential segregation were constructed using information from the Geographic Register on Demographic Statistical Areas (DeSO; demografiska statistikområden), created and maintained by Statistics Sweden (Statistics Sweden, 2023). Sweden has been subdivided into 5,984 DeSO units, which consider geographic characteristics such as streets, rivers, and railways, as well as urbanicity and electoral districts, and are comprised of approximately 700–2,700 inhabitants each. The DeSO measure first became available in 2018, and was applied retrospectively to the data from 2004 to ensure that all DeSOs contained a sufficient number of residents to assess residential segregation and associations with mortality. All measures were calculated annually at the DeSO level for the entire duration of follow-up and categorized into quintiles (Q1-Q5). Quintiles were used to ensure comparability across time, as the measures are not margin free (Elbers, 2023).

To formulate each of the measures, the global level area units (metropolitan regions of Stockholm, Gothenburg, Malmö) were subdivided into N local level area units (DeSOs), indexed by n = 1 … N. Native- and foreign-born residents within each DeSO were indexed by i (migrant) and s (native-born) or, when origin was accounted for, by j (Global North migrants), k (Global South migrants), and s (native-born). T comprised the total number of individuals in the metropolitan region, with t_n_ the total number of individuals in local area unit n, t_i_ the total number of migrants in the metropolitan area, and t_ni_ the number of migrants in local area unit n. Consequently, the proportion (π) of migrants was denoted by π_i_ = t_i_/T, the proportion of all individuals residing in area unit n was denoted by π_n_ = t_n_/T, and the proportion of migrants in area unit n denoted as π_ni_ = t_ni_/t_n_.

#### 2.2.1 Composition measure: migrant density

Measures of composition assess the proportion of population group(s) living in specific residential areas. Migrant residential composition was measured in terms of migrant density, and was calculated using the proportion of migrants residing in each DeSO, ranging from 0 to 1, where higher values indicated higher migrant density within a given DeSO. The density of migrants (i) in DeSO (n) can be expressed as: $\mathrm{Migrant}\;\mathrm{density}=\pi_{n,i}=\frac{t_{n,i}}{t_{n}}$. When further defined by origin, migrant density among those from the Global North (j) is expressed as: $\pi_{n,j}=\frac{t_{n,j}}{t_{n}}$, and for migrants from the Global South (k) expressed as: $\pi_{n,k}=\frac{t_{n,k}}{t_{n}}$.

#### 2.2.2 Interaction measure: Local Isolation/Exposure (Lex/Is) metrics

Measures of interaction assess the likelihood of interaction between different population groups within specified residential areas, and thus reflect the probability of a minority individual sharing a residential space with a minority or majority-population individual. The Local Exposure and Isolation (LEx/Is) metrics (Bemanian & Beyer, 2017) were used to assess the probability of interaction between native- and foreign-born DeSO residents. These metrics were developed in order to more accurately measure interaction between groups at the local level by standardizing the probability of interaction between groups (Bemanian & Beyer, 2017). Within each DeSO, local migrant isolation reflected the probability of interaction between two migrant individuals, while local migrant-native exposure reflected the probability of interaction between a migrant and native-born. Values of zero indicated that the probability of interaction within a given DeSO was equivalent to the probability of interaction in a perfectly mixed, i.e., not at all segregated, metropolitan area. Values greater than zero indicated a greater likelihood of interaction within the DeSO than within the metropolitan area, and values less than zero indicated a lower likelihood of interaction within the DeSO than in the metropolitan area. The Lex/Is metrics are non-linear functions of the proportion of migrant residents within each DeSO. Formally, local migrant isolation is expressed as ${Isolation}_{i,n}=\log\left(\frac{\pi_{n,i}\times \pi_{n,i}}{1-\pi_{n,i}\times \pi_{n,i}}\right)-\log\left(\frac{\pi_{i}\times \pi_{i}}{1-\pi_{i}\times \pi_{i}}\right)$; and local migrant-native exposure as: ${Exposure}_{i,s,n}=\log\left(\frac{\pi_{n,i}\times \pi_{n,s}}{1-\pi_{n,i}\times \pi_{n,s}}\right)-\log\left(\frac{\pi_{i}\times \pi_{s}}{1-\pi_{i}\times \pi_{s}}\right)$*.* Throughout the remainder of the text, local migration isolation will be referred to as “migrant isolation” or “isolation”, while local migrant-native exposure will be referred to as “migrant exposure” or “exposure”.

#### 2.2.3 Distribution measure: The mutual information index

Measures of distribution assess the extent to which population groups are evenly or unevenly spread across specific residential areas. Distributional measures use the proportion of population groups at the global level as a reference for assessing the extent to which segregation at the local level is present. In other words, a non-segregated local area is defined as one with an even distribution, containing the same distribution of population groups as that observed at the global level. The mutual information index (Mora & Ruiz-Castillo, 2011; Theil & Finizza, 1971) was used to assess the evenness of the distribution of migrant and native-born residents within each DeSO. The mutual information index is an entropy-based measure that captures deviation from an idealized reference distribution, (i.e., one in which all DeSOs have the same proportion of migrants as the metropolitan area as a whole). The metropolitan areas of Stockholm, Gothenburg, and Malmö were used as the global levels from which the idealized reference distributions were derived. The mutual information index was calculated as weighted sums of the relative entropy of migrant group proportions in DeSOs. Values of zero reflected instances in which the proportions of migrants in a given DeSO and metropolitan region were equivalent. Values above zero reflected deviation from the idealized reference distribution, with higher values indicating a greater degree of unevenness. The mutual information index has been suggested to improve upon other entropy-based measures of evenness, such as the spatial information (H) index, since it can be extended to multiple groups, and has a clearer interpretation at the local level (Elbers, 2021; Roberto, 2016). While other entropy-based measures can include negative values at the local level, the mutual information index begins at zero, whereby group proportions are equivalent at the local and global levels, and increase monotonically as proportions deviate from this reference distribution. The mutual information index (M) in each DeSO (n) can be expressed as: $M_{n}=\sum_{g}\pi_{\text{ng}}\;\ln\frac{\pi_{\text{ng}}}{\pi_{g}}$. When M is measured by nativity, the summation over g = {i, s}, with migrants (i) and the native-born (s). When classified by origin, the summation over g = {j, k, s}, with migrants from the Global North (j), migrants from the Global South (k), and the native-born (s).

### 2.3 Outcome and covariates

The outcome was all-cause mortality, which was identified using the Cause of Death Register. Covariates were derived from the Longitudinal Integrated Database for Health Insurance and Labor Market Studies (LISA). With the exception of sex (men and women), individual-level covariates were modeled as varying over time, with assessment every five years, including: age (assessed as a categorical variable with five-year age bands), as well as education level, income, and labor market status. Level of attained education was measured according to the International Standard Classification of Education (ISCED) codes and categorized into ‘*primary’*, ‘*secondary*’, and ‘*post-secondary*’. Disposable household income was equivalized and grouped into terciles. Labor market status was dichotomized as ‘*employed’* or *‘not in employment’*. Missingness on time-varying covariates entailed exclusion of follow-up time. A measure of area-level socioeconomic deprivation was also constructed for each DeSO and modeled as an annual time-varying covariate. The measure was constructed by combining information on the proportion of adult residents in each DeSO who were in relative poverty (defined as equivalized disposable household income lower than 60% of the population median income), not in employment, received social assistance benefits, and had lower levels of education (ISCED level 2 or below). Principal component analysis was used to create an index of socioeconomic deprivation, divided into deciles, ranging from the least deprived (decile 1) to the most deprived (decile 10). Information on how principal component analysis was used to construct this index can be found in Supplementary File S1.

### 2.4 Statistical analysis

Multi-level Poisson regression models in which the data were grouped by DeSO were used to estimate associations between the migrant density, isolation, exposure, and evenness indices and all-cause mortality. The logarithm of the follow-up time in person-years was included as the offset. We tested for overdispersion and found that the assumptions regarding the equality of the mean and variance were not violated. Cross-nested models were used to account for changes in geographic residence between DeSO units within a metropolitan region during follow-up. Median rate ratios (MRR) were calculated to estimate general contextual effects (Austin et al., 2018). Multiplicative interaction terms for each segregation measure and nativity/origin were used to assess for modification by migrant background, and likelihood ratio tests showed that the interaction terms were statistically significant and improved model fit. Clustered robust standard errors were calculated to account for correlations between observations. Our modeling strategy comprised a three-stage adjustment approach: A minimally adjusted model (age and sex; Model 1); a model further adjusted for individual-level covariates (Model 2); and a fully adjusted model including all individual- and area-level covariates (Model 3). All models were run on aggregated data for computational purposes (i.e., categorical specifications). In all analyses, the lowest quintile of residential segregation served as the reference group.

All data management and statistical analyses were conducted in R version 4.2.2.

## 3 Results

Table 1 shows descriptive statistics, crude and age and sex standardized mortality rates, and proportions of person-time across individual-level covariates by metropolitan area of residence and migration background. Migrants, particularly individuals from the Global South, were younger than the native-born and generally also showed lower mortality rates. Higher proportions of person-time spent in low education, low income, and not in employment were observed among migrants than the native-born. Supplementary Table S1 shows proportions of person-time across the measures of residential segregation and local socioeconomic deprivation by metropolitan area of residence and migration background. Proportions of person-time in DeSOs characterized by greater socioeconomic deprivation were higher among migrants, particularly those from the Global South, than among the native-born. A positive gradient in the proportion of person-time across quintiles of migrant density, isolation, and exposure was also observed among migrants. Higher proportions of person-time in areas with the highest unevenness (Q5) were also seen among migrants, although a gradient was not observed across quintiles. The spatial mapping of the segregation measures across DeSOs in each metropolitan area is shown in Supplementary Figs. S1 and S2. The maps show high degrees of correlation between the migrant density, isolation, and exposure measures. This was also confirmed by a correlation analysis of the segregation measures (see Supplementary File S2 for a correlation matrix).

**Table 1:** Descriptive statistics and study population person-time, number of deaths, and mortality rates by metropolitan area of residence, nativity, and region of origin.

Stockholm	Native-born	All Migrants	Global North	Global South
**N (%)**	1,540,844 (71.3)	621,224 (28.7)	309,164 (14.3)	311,747 (14.4)
**Person-years (PY)**	15,423,930	5,052,515	2,503,209	2,547,831
**Deaths (N)**	164,676	35,278	27,975	7297
**Mortality rate**^a^	1067.7	698.2	1117.6	286.4
**Std. Mortality rate**^b^	985.0 (980.2–989.7)	931.3 (921.4–941.4)	989.9 (978.2–1001.8)	755.2 (735.2–775.7)
**Mean age (years)**	46.4	43.8	47.9	39.8
**Sex (N (%))**				
Men	758,481 (49.2)	310,453 (50.0)	149,173 (48.3)	161,089 (51.7)
Women	782,363 (50.8)	310,771 (50.0)	159,991 (51.7)	150,658 (48.3)
**Education (PY (%))**				
Primary	3,566,335 (23.1)	1,744,405 (34.5)	755,709 (30.2)	988,049 (38.8)
Secondary	5,844,873 (37.9)	1,600,139 (31.7)	837,662 (33.5)	762,119 (29.9)
Post-secondary	6,012,722 (39.0)	1,707,971 (33.8)	909,838 (36.3)	797,663 (31.3)
**Income (PY (%))**				
Tercile 1 (lowest)	2,573,145 (16.7)	2,223,866 (44.0)	932,454 (37.3)	1,290,424 (50.6)
Tercile 2	8,220,830 (53.3)	2,187,673 (43.3)	1,150,843 (46.0)	1,036,439 (40.7)
Tercile 3 (highest)	4,629,955 (30.0)	640,976 (12.7)	419,912 (16.8)	220,968 (8.7)
**Labor market status (PY (%))**				
Employed	9,867,895 (64.0)	2,566,705 (50.8)	1,292,125 (51.6)	1,273,888 (50.0)
Not in employment	5,556,035 (36.0)	2,485,810 (49.2)	1,211,084 (48.4)	1,273,943 (50.0)

Gothenburg	Native-born	All Migrants	Global North	Global South
**N (%)**	761,287 (77.5)	220,857 (22.5)	113,933 (11.6)	106,870 (10.9)
**Person-years (PY)**	7,527,799	1,797,244	963,215	833,711
**Deaths (N)**	85,835	13,472	11,409	2063
**Mortality rate**^a^	1140.2	749.6	1184.5	247.4
**Std mortality rate**^b^	1062.2 (1055.1–1069.4)	1065.8 (1047.0–1084.9)	1132.0 (1110.6–1153.7)	794.2 (752.9–837.7)
**Mean age (years)**	46.7	43.6	47.9	38.7
**Sex (N (%))**				
Men	375,496 (49.3)	112,342 (50.9)	57,030 (50.1)	55,289 (51.7)
Women	385,791 (50.7)	108,515 (49.1)	56,903 (49.9)	51,581 (48.3)
**Education (PY (%))**				
Primary	1,944,512 (25.8)	630,660 (35.1)	310,788 (32.3)	319,716 (38.3)
Secondary	2,895,631 (38.5)	612,562 (34.1)	352,936 (36.6)	259,540 (31.1)
Post-secondary	2,687,656 (35.7)	554,022 (30.8)	299,491 (31.1)	254,455 (30.5)
**Income (PY (%))**				
Tercile 1 (lowest)	1,414,033 (18.8)	806,048 (44.9)	353,060 (36.7)	452,782 (54.3)
Tercile 2	3,949,061 (52.5)	758,223 (42.2)	449,417 (46.7)	308,727 (37.0)
Tercile 3 (highest)	2,164,705 (28.8)	232,973 (13.0)	160,738 (16.7)	72,202 (8.7)
**Labor market status (PY (%))**				
Employed	4,695,544 (62.4)	828,519 (46.1)	464,538 (48.2)	363,863 (43.6)
Not employed	2,832,259 (37.6)	968,725 (53.9)	498,677 (51.8)	469,848 (56.4)

Malmö	Native-born	All Migrants	Global North	Global South
**N (%)**	512,825 (72.0)	199,558 (28.0)	118,847 (16.7)	80,537 (11.3)
**Person-years (PY)**	4,996,917	1,526,314	941,266	584,173
**Deaths (N)**	61,774	10,130	8740	1386
**Mortality rate**^a^	1236.2	663.7	928.5	237.3
**Std mortality rate**^b^	1105.8 (1097.1–1114.5)	1073.2 (1051.4–1095.4)	1118.9 (1094.9–1143.4)	826.1 (773.2–882.6)
**Mean age (years)**	47.3	42.3	45.1	37.9
**Sex (N (%))**				
Men	251,101 (49.0)	100,833 (50.5)	59,090 (49.7)	41,646 (51.7)
Women	261,724 (51.0)	98,725 (49.5)	59,757 (50.3)	38,891 (48.3)
**Education (PY (%))**				
Primary	1,311,693 (26.3)	549,510 (36.0)	309,908 (32.9)	239,178 (40.9)
Secondary	1,863,606 (37.3)	481,107 (31.5)	325,560 (34.6)	155,383 (26.6)
Post-secondary	1,821,618 (36.5)	495,697 (32.5)	305,798 (32.5)	189,612 (32.5)
**Income (PY (%))**				
Tercile 1 (lowest)	779,109 (15.6)	715,887 (46.9)	380,619 (40.4)	334,592 (57.3)
Tercile 2	2,708,592 (54.2)	627,684 (41.1)	421,363 (44.8)	206,157 (35.3)
Tercile 3 (highest)	1,509,216 (30.2)	182,743 (12.0)	139,284 (14.8)	43,424 (7.4)
**Labor market status (PY (%))**				
Employed	2,893,883 (57.9)	593,127 (38.9)	393,308 (41.8)	199,661 (34.2)
Not employed	2,103,044 (42.1)	933,187 (61.1)	547,958 (58.2)	384,512 (65.8)

a Crude mortality rates/100,000 person-years.

b Age and sex standardized mortality rates/100,000 person-years with 95% confidence intervals.

**Fig. 1:**
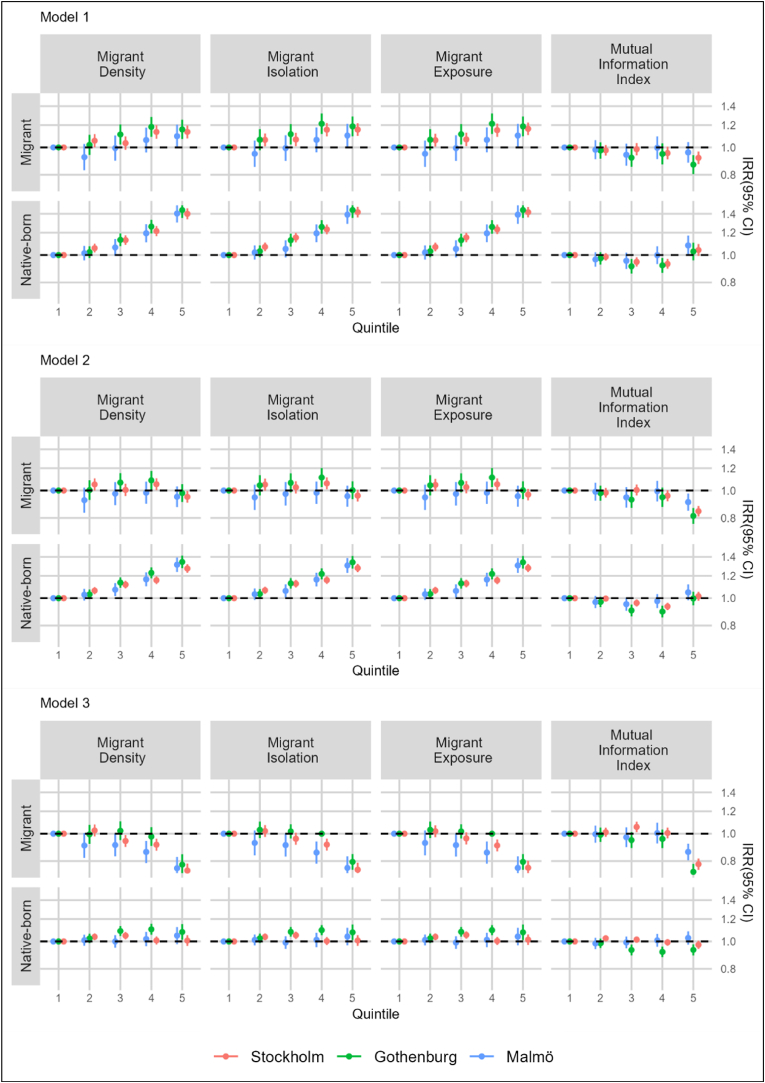
Incidence Rate Ratios (IRR) for all-cause mortality associated with migrant residential segregation among native-born and migrant residents of Stockholm, Gothenburg, and Malmö. Model 1: adjusted for age and sex. Model 2: adjusted for age, sex, education level, disposable household income, and labor market status. Model 3: adjusted for age, sex, education level, disposable household income, labor market status, and area-level socioeconomic deprivation. Note: In order to enhance readability some confidence intervals have been truncated at upper and lower limits.

Fig. 1 shows incidence rate ratios (IRR) with 95% confidence intervals (CI) for all-cause mortality associated with indices of migrant residential segregation among native-born and migrant residents of each metropolitan area (see Supplementary Table S2 for point estimates and median rate ratios in tabular form). Moderate positive gradients in the risk of mortality were observed across quintiles of migrant density, isolation, and exposure, among both the native-born and migrants (Model 1). Adjustment for individual-level socioeconomic factors (Model 2) largely accounted for the increased mortality risks observed among migrants, and additional adjustment for neighborhood socioeconomic deprivation (Model 3) largely explained the elevated risk estimates among the native-born. Among migrants, moderate decreased risk estimates for mortality (ranging from IRR 0.74–0.79 across metropolitan areas) were observed in fully adjusted models within the highest quintiles of migrant density, isolation and exposure across all metropolitan areas. Moderate decreased mortality risk estimates were also observed among migrants within the highest quintiles of unevenness (ranging from IRR 0.73–0.86). There was little variation in mortality risk estimates across quintiles of evenness among the native-born, with the exception of Gothenburg, whereby slightly decreased IRRs were observed in Q2-Q5 (IRRs ranged from 0.92 to 0.93). Median rate ratios, which reflected the median relative change in mortality rates when comparing individuals with the same covariate characteristics from randomly selected neighborhoods, ranged from 1.12 to 1.13 across all measures and metropolitan areas in fully adjusted models.

**Fig. 2:**
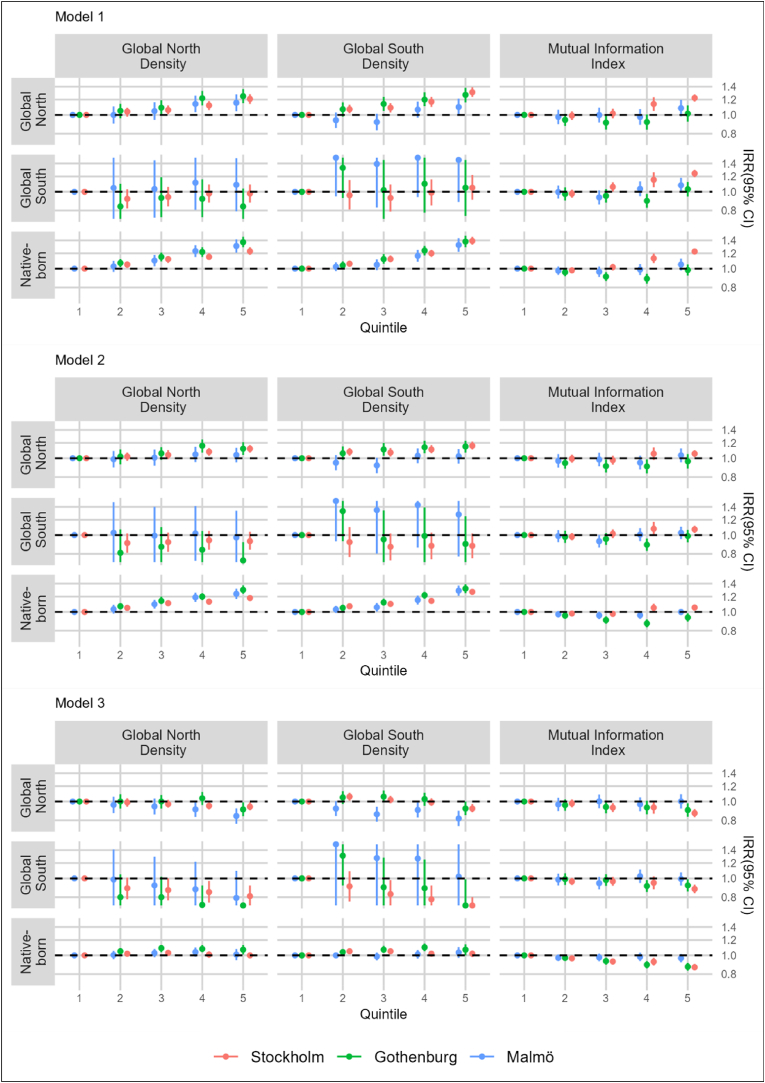
Incidence Rate Ratios (IRR) for all-cause mortality associated with migrant density and migrant residential segregation among native-born and migrant residents of Stockholm, Gothenburg, and Malmö. Model 1: adjusted for age and sex. Model 2: adjusted for age, sex, education level, disposable household income, and labor market status. Model 3: adjusted for age, sex, education level, disposable household income, labor market status, and area-level socioeconomic deprivation. Note: In order to enhance readability some confidence intervals have been truncated at upper and lower limits.

Fig. 2 shows incidence rate ratios (IRR) with 95% confidence intervals (CI) for all-cause mortality by quintiles of migrant density and unevenness, but further distinguished among migrants by Global North and Global South origin (see Supplementary Table S3 for point estimates and median rate ratios in tabular form). Results for migrant isolation and migrant exposure which accounted for origin were qualitatively similar to those observed for migrant density, and are available upon request. The overall patterns of findings were similar to those observed for the analyses which used binary (i.e., migrant/native-born) measures of migrant density and evenness, with some exceptions. In minimally adjusted analyses (Model 1), positive gradients in the risk for mortality were observed only among the native-born and migrants from the Global North across measures of Global North density and Global South density. Modest gradients in mortality risk by unevenness were observed in Stockholm among the native-born and migrants from the Global North and Global South (Model 1); these gradients largely attenuated with adjustment for individual-level socioeconomic indicators (Model 2).

In fully adjusted analyses (Model 3), modest decreased IRRs of mortality (Q5 vs Q1) were observed among migrants from the Global North across measures of Global North density, Global South density, and unevenness in all metropolitan areas, excepting those who resided in the highest quintile of unevenness in Malmö. Among migrants from the Global South, moderate decreased mortality risk estimates were observed in the highest quintiles of Global North and Global South density in Stockholm and Gothenburg. Among the native-born, substantive differences in mortality across the measures were largely not observed, although slightly decreased mortality risks were seen across quintiles of unevenness in Stockholm and Gothenburg (Q2-Q5 in Stockholm; Q3-Q5 in Gothenburg), and slightly elevated mortality risks were observed among the native-born in Gothenburg across quintiles of Global North and Global South density (Q2-Q5). Median rate ratios in fully adjusted models did not differ from those observed in analyses which did not account for Global North or Global South origin.

## 4 Discussion

This register based, longitudinal study provides new insights for understanding the role of migrant residential segregation in relation to health, a topic which has been understudied in the Swedish context. We employed local measures of composition (migrant density), interaction (isolation/exposure), and distribution (evenness) to thoroughly assess the risk of all-cause mortality by migrant residential segregation within Sweden's largest metropolitan regions. Within study population subgroups of migrants and the native-born, patterns of association were largely consistent in direction and magnitude across measures of migrant density, isolation, and exposure in all three metropolitan regions; however, patterns diverged when segregation was measured in terms of evenness. Differential patterns of association between mortality and residential segregation were also observed between migrants and the native-born. In fully adjusted analyses, moderately decreased mortality risks were observed among migrants in residential areas characterized by higher levels of migrant density, isolation, and exposure, suggesting possible protective health effects. Modest decreased mortality risks were also observed among migrants in areas with the highest degree of unevenness. Among the native-born, mortality risk differences by migrant residential segregation were largely negligible.

### 4.1 Comparison of migrant residential segregation indices

Previous research has suggested that measures of residential composition versus measures of segregation can yield different patterns of association in relation to health outcomes (Acevedo-Garcia & Lochner, 2003; White & Borrell, 2011). For instance, a review of research on ethnic and racial segregation and health in the US showed lower mortality risks among Blacks in studies that used measures of local Black composition, whereas studies which employed segregation measures showed higher mortality risks among Blacks (White & Borrell, 2011). However, our results showed similar patterns of association across measures of migrant density, isolation, and exposure, within both the migrant and native-born study populations. The similarity of our findings across these measures can likely be largely attributed to the fact that our migrant isolation and exposure measures were monotonically increasing functions of migrant density at the local level. As such, despite conceptual and measurement distinctions, our relative indices of local migrant density, isolation, and exposure were all directly related to the proportion of migrant residents within each DeSO neighborhood. The strong correlation between the isolation and exposure measures also entailed that we were unable to determine whether the observed associations with mortality were due to isolation or exposure, and the role of each of these aspects of segregation remains unclear. In addition, although the measures of migrant isolation and exposure considered the global proportion of migrants at the metropolitan area level, this did not appear to impact the pattern of associations found. This could be due to our use of relative measures, necessary for our longitudinal analysis of migrant residential segregation, whereby the global proportions of migrants were included in the migrant isolation and exposure indices in the same way across quintiles. Thus, local measures of isolation and exposure may be better suited to capture interaction when employed as absolute measures of interaction at one point in time. However, additional research is needed to investigate this further, and to continue developing local measures of isolation and exposure that are appropriate for longitudinal studies.

The mutual information index, used to assess the evenness dimension of segregation, is an entropic measure that reflects the extent to which the distribution of individuals across residential areas deviates from an idealized perfectly “mixed” distribution of residents. As the mutual information index is concerned with the evenness of distributions, DeSO neighborhoods with both the lowest and highest proportions of migrant residents were classified in the highest quintile of unevenness. This lack of distinction between high and low proportions of migrant residents may explain why clear mortality gradients by unevenness were not observed, and also why patterns of findings diverged from those observed for migrant density, isolation, and exposure; for example, in minimally adjusted models, positive gradients were observed for migrants and the native-born across measures of migrant density, isolation, and exposure, whereas clear gradients were not observed among migrants and the native-born when the mutual information index was employed, although a slightly decreased mortality risk was seen among migrants in the most segregated (Q5) areas. Relatedly, although measures of the evenness dimension of segregation have been widely utilized, evenness may not capture the aspects of segregation that are the most relevant for health (Acevedo-Garcia et al., 2003), such as neighborhood quality or socioeconomic deprivation. Despite the utility of the mutual information index as a local measure of evenness (Mora & Ruiz-Castillo, 2011), our findings suggest that evenness may not be particularly informative for understanding the complex association between migrant residential segregation and the risk of all-cause mortality in the Swedish context.

### 4.2 Comparison of native-born and migrant groups

Differential patterns of association between mortality risks and migrant residential segregation were observed between native-born and migrant groups. While positive gradients in mortality risk were observed among native-born residents in areas with high migrant density, isolation, and exposure—both in minimally adjusted models and models adjusted for individual-level socioeconomic factors—these associations were largely negligible in fully adjusted models that accounted for both individual- and contextual-level socioeconomic factors. Swedish urban residential areas with higher proportions of migrants tend to be more economically deprived compared to those with higher proportions of native-born residents (Malmberg & Clark, 2021). Thus, the attenuation of the risk gradient suggests that neighborhood economic deprivation may have been a significant contributor to the excess mortality risks observed among the native-born. An exception to this overall pattern was observed in Gothenburg, whereby modest elevated mortality risks in relation to measures of migrant density, isolation, and exposure remained across quintiles three through five, and modest decreased risks of mortality in evenness quintiles three through five were observed among the native-born in fully adjusted models. However, a lack of previous Swedish studies on relationships between residential segregation and mortality makes interpretation of these findings difficult. Associations between evenness and mortality were largely negligible among the native-born in the other metropolitan areas, and taken together, the overall findings suggest a minimal role of migrant residential segregation in relation to mortality risks among the native-born after adjustment for individual- and contextual-level socioeconomic factors.

Among migrants, modest positive gradients in mortality risk were observed across quintiles of migrant density, isolation, and exposure, which largely attenuated after adjustment for individual-level socioeconomic factors. In fully adjusted analyses, moderate decreased mortality risks were observed among migrants residing in the highest quintiles of migrant density, isolation, and exposure, in all metropolitan areas. The roles of isolation and exposure in these associations could not be differentiated from each other due to strong correlation between these measures. However, the findings for migrant density are in line with previous studies that have shown protective health effects of ethnic and migrant density (Becares et al., 2018; Dykxhoorn et al., 2020; Hutchinson et al., 2009; Inagami et al., 2006). Psychosocial factors related to local social environments, including potential protective and buffering effects of increased social support and social inclusion and fewer experiences of racism and discrimination (Becares et al., 2009; Pickett & Wilkinson, 2008), may help to explain the decreased mortality risks observed. However, in contrast to findings from other Nordic contexts (Schofield et al., 2024), our results did not consistently reveal stronger effects when migrant density by origin was considered. Although decreased mortality risks associated with residence in areas of higher migrant density were slightly more pronounced among migrants from the Global South than among migrants from the Global North, migrant density by origin did not appear to influence the magnitude of the decreased mortality risks observed. Rather, decreased mortality risks were of similar magnitudes within origin groups regardless of whether they resided in areas of high Global North or Global South density. This suggests that residence in areas with high migrant density more generally may also confer protective effects for health. Recent findings have suggested that some of the health benefits associated with co-ethnic density specifically may be overstated, and may actually extend to other groups residing in areas with greater ethnic diversity (Seo & Jivraj, 2024). For instance, ethnic minority migrants residing in migrant dense neighborhoods may experience similar protections from, for example, experiences of racism or discrimination as do those who reside in areas of high co-ethnic density more specifically. Similarly, forms of community support (e.g., from neighbors, local social institutions) in neighborhoods with higher ethnic density are likely to extend beyond specific ethnic groups (Seo & Jivraj, 2024). This may be particularly relevant for the Swedish context, as migrant dense neighborhoods are multi-ethnic in nature, comprised of residents from many different countries of origin (Andersson, 2007).

Conversely, our broad categorization of migrant density by Global North and Global South origin may have entailed too much heterogeneity for risk differences to be observed. While our measure may have captured broad differences in experiences of social integration or marginalization between these groups, it may not have sufficiently reflected the sense of belonging or shared language, cultural values, and traditions that can exist between persons from the same country or region. Despite our use of total population register data, we were unable to assess more specific categories of origin at the DeSO level due to the diversity of the migrant population in Sweden by origin and associated statistical power limitations in relation to mortality cases. Future studies should investigate the roles of neighborhood migrant density and own-region density in relation to cause specific mortality and other physical health outcomes further, as much of the existing literature on density effects has specifically examined relationships between co-ethnic density and mental health.

Fully adjusted analyses also showed decreased mortality risks among migrants residing in areas with the highest levels of unevenness. These decreased risks were likewise observed in analyses that accounted for Global North and Global South origin, with the exception of Malmö. However, the decreased mortality risks observed in relation to unevenness were more modest when compared to those for density in that, similar to findings for the native-born, gradients in mortality risk were not evident. As such, these results also point to a limited role of evenness in influencing the risk of mortality among migrants.

### 4.3 Strengths and limitations

The primary strength of this study was the use of a multilevel analysis framework applied to longitudinal data, which enabled us to measure different aspects of local residential segregation by nativity and origin over time, and to account for potential local level changes in segregation throughout the study period, as well as movement between residential neighborhoods within metropolitan areas. To our knowledge, this is the first study in Sweden to longitudinally compare measures of migrant segregation on a local level in relation to mortality. However, our study was also tempered by several limitations that should be considered. First, as previously mentioned, the heterogeneity of the migrant population in Sweden entailed the use of broad categories of origin in our analysis, which limited the extent to which we could investigate relationships between mortality and own-region of origin density. The younger demographic profile of the migrant population from the Global South entailed lower crude mortality rates in this group, and thus also limited the extent to which we could disaggregate our analyses by origin. Second, our analysis focused only on the three largest metropolitan areas of Sweden, rather than the entire country. Although migrants often tend to settle in metropolitan areas (Diaz Ramirez et al., 2018), particularly during the initial years following migration, it remains unclear how patterns of segregation may be related to mortality risk in less densely populated areas. Third, the strong correlation between our measures of isolation and exposure limited the interpretation of these findings, as we could not determine whether the observed associations with mortality were due to isolation or exposure. In addition, many adult individuals spend substantial proportions of their daily lives away from their residential neighborhoods for work, studies, or leisure. However, we were not able to account for time spent away from residential areas, or the extent to which individuals may experience forms of discrimination or marginalization in other contexts, which can also have health implications. There may also have been health selection effects that were unaccounted for in the current study. Many migrants residing in high income countries demonstrate a mortality advantage relative to the native-born population (Aldridge et al., 2018; Shor & Roelfs, 2021). Although this health advantage has been shown to diminish with longer residence in the destination country (Wallace et al., 2019) and to also vary by age (Guillot et al., 2018) and origin (Shor & Roelfs, 2021), the decreased mortality risks observed among migrants in areas with greater migrant composition may partially reflect this mortality advantage. However, in our analyses that accounted for origin, we adjusted for differences in mortality by Global North or Global South origin. These findings did not differ substantially from those in the main analysis, although a slightly more substantive mortality advantage was observed in migrants from the Global South compared to migrants from the Global North. The potential health and socioeconomic selection effects in and out of neighborhoods with different socioeconomic and demographic characteristics further limits the interpretation of our findings. Additional research is needed to better elucidate potential mechanisms behind the decreased mortality risks which were observed, and to also more closely examine the potential roles of the migrant mortality advantage, duration of residence, and health and socioeconomic selection effects in and out of neighborhoods. Additionally, the duration of our follow-up period was limited to 2016, and as such, our study does not reflect individuals who have relocated to Sweden in the past several years. However, given that the migrant population in Sweden is diverse by origin and comprises a substantial proportion of the total population, it is unlikely that our overall pattern of findings would change with an extended follow-up period. Finally, the 95% confidence intervals for our risk estimates should be interpreted with some caution, as multiple comparisons of the segregation measures in relation to mortality were made. However, after applying a Bonferroni correction, the patterns of our findings largely remained robust (see Supplementary Tables 2 and 3 which denote the statistical significance levels of the point estimates at p < 0.0125).

## 5 Conclusions

Although Swedish migration and integration policies have recently become more restrictive (European Commission, 2024), for several decades these policies have been amongst the most generous in Europe. Despite this, neighborhoods with high concentrations of migrant residents persist. A myriad of factors contribute to the residential sorting of migrants (e.g., housing policy and de-regulated housing markets, selective migration) but a key explanation relates to the socioeconomic and labor market position of migrants, which tends to be more disadvantaged than the native-born (Dunlavy, 2017; Rostila & Hjern, 2018; Swedish Agency for Work Environment Expertise, 2024). The high correlation between migrant and income residential segregation (Hedman & Andersson, 2016; Malmberg & Clark, 2021) suggests that integration policies have not fully succeeded in facilitating the economic and spatial integration of migrants. At the same time, residence in migrant dense areas may foster social support and a sense of community, and serve to buffer against harmful experiences of racism or discrimination. As such, the implementation of wide-ranging policies aimed at fostering economic inclusion and combating income inequalities, rather than migrant segregation per se, may be more effective measures for combating concentrated socioeconomic disadvantage and promoting integration.

This study provides new evidence from the Swedish context on relationships between migrant residential segregation and risk of mortality in both native-born and migrant populations. After accounting for individual- and area-level socioeconomic factors, migrants residing in areas with higher proportions of migrants, irrespective of origin, demonstrated moderate reduced mortality risks, suggesting potential protective effects. In contrast, associations between migrant residential segregation and mortality were largely not observed among the native-born. Increased understanding of the mechanisms and pathways through which different aspects of segregation may influence health can help to inform strategies for fostering social, economic, and residential integration, and ultimately improving population health.

## CRediT authorship contribution statement

**Agneta Cederström:** Writing – review & editing, Writing – original draft, Visualization, Methodology, Funding acquisition, Formal analysis, Data curation, Conceptualization. **Andrea Dunlavy:** Writing – review & editing, Writing – original draft, Methodology, Funding acquisition, Conceptualization.

## Ethical approval

Ethical approval for the study was granted by the Regional Ethical Review Board inStockholm, Sweden (decision no. 2017/716–31/5).

## Funding

The authors acknowledge funding for this research from 10.13039/501100006636The Swedish Research Council for Health, Working Life and Welfare (FORTE; grant number 2016-07128).

## Declaration of competing interest

The authors declare that they have no known competing financial interests or personal relationships that could have appeared to influence the work reported in this paper.

## Data Availability

The authors do not have permission to share data.
