# Supplementary material for: A matter of measurement? A Swedish register-based study of migrant residential segregation and all-cause mortality
Source: SSM Popul Health. 2025 Mar 27;30:101793. doi: 10.1016/j.ssmph.2025.101793 (PMC12005324; doi:10.1016/j.ssmph.2025.101793)
Supplement: Multimedia component 1 [file mmc1.docx]

Supplementary File S1. Information on the principal components analysis used to calculate the local area-level indicator of socioeconomic deprivation.

A measure of area-level socioeconomic deprivation was developed to capture the relative socioeconomic conditions in each DeSO (Demographic Statistical Area), modeled as an annual time-varying covariate. This measure incorporated four indicators of socioeconomic conditions:

1. **Relative Poverty**: The proportion of adult residents in each DeSO with equivalized disposable household incomes below 60% of the population median income. This measure aligns with established definitions of relative poverty.
2. **Not in employment**: The proportion of adult residents not engaged in employment, capturing labor market exclusion as a component of socioeconomic deprivation.
3. **Social Assistance Benefits**: The proportion of adult residents receiving social assistance benefits, serving as a proxy for use of welfare services due to economic or other types of hardship.
4. **Educational Attainment**: The proportion of adult residents with low educational attainment, defined as having completed at most ISCED (International Standard Classification of Education) Level 2, which includes lower secondary education or below.

These proportions were calculated separately for each indicator to provide a consistent, comparable basis for measuring socioeconomic conditions across DeSOs. To construct the deprivation index, Principal Component Analysis (PCA) was employed, following the standardization of the proportions of the indicators to have a mean of zero and a standard deviation of one. After scaling the variables, the covariance matrix was calculated, which measured the extent to which pairs of variables varied together. Specifically, the covariance matrix was a symmetric 4x4 matrix where each element represented the covariance between any two indicators across DeSOs (variance across diagonal elements of the matrix). From this matrix, eigenvectors (which represent an orthogonal basis along the directions of maximum variance) and eigenvalues (the amount of variance explained by each direction) were extracted. Each eigenvector represented a principal component, with its elements defining the contribution of each variable to that component. The first principal component (PC1), characterized by the eigenvector associated with the largest eigenvalue, was selected as the deprivation index because it captured the greatest proportion of shared variance across the indicators. This proportion of the shared variance ranged from ≈ 70-80% across the years considered. This approach synthesized the indicator variables into a single, interpretable measure, reflecting patterns of socioeconomic deprivation across DeSOs.

The resulting index was further standardized for ease of interpretation and divided into deciles, ranging from the least deprived (decile 1) to the most deprived (decile 10). The use of deciles provided a granular representation of area-level socioeconomic deprivation, enabling clear comparisons across time and geographic areas.
