# Supplementary material for: A matter of measurement? A Swedish register-based study of migrant residential segregation and all-cause mortality
Source: SSM Popul Health. 2025 Mar 27;30:101793. doi: 10.1016/j.ssmph.2025.101793 (PMC12005324; doi:10.1016/j.ssmph.2025.101793)
Supplement: Multimedia component 3 [file mmc3.docx]

Supplementary Figure S1. Spatial mapping of measures of migrant density, migrant isolation, migrant exposure and the two-group Mutual Information Index* across metropolitan areas (2014).


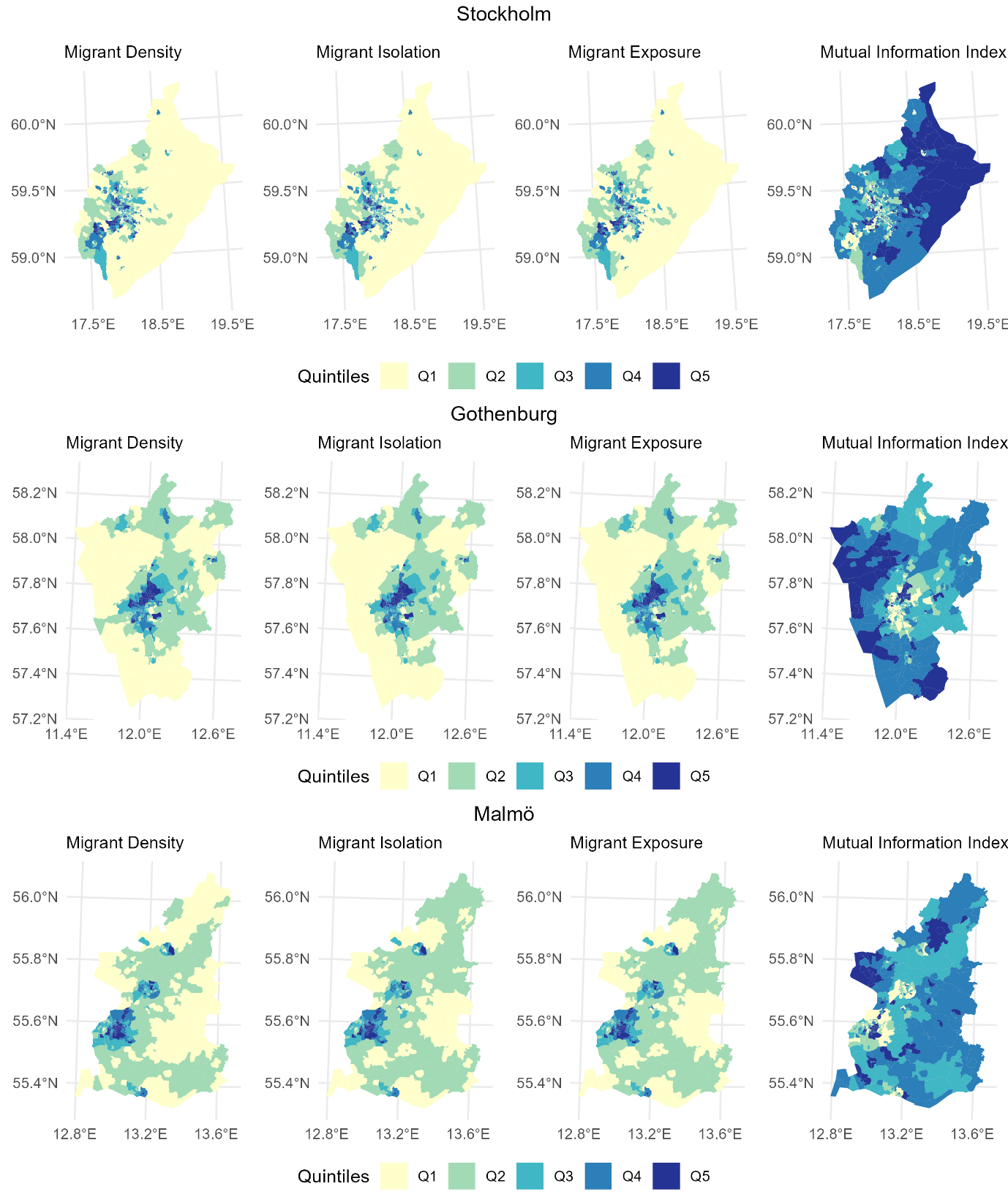


*The two group Mutual Information Index included groups categorized as native-born and foreign-born.
