# Supplementary material for: A matter of measurement? A Swedish register-based study of migrant residential segregation and all-cause mortality
Source: SSM Popul Health. 2025 Mar 27;30:101793. doi: 10.1016/j.ssmph.2025.101793 (PMC12005324; doi:10.1016/j.ssmph.2025.101793)
Supplement: Multimedia component 4 [file mmc4.docx]

Supplementary Figure S2. The spatial mapping of measures of Global North Density, Global South Density and the three-group Mutual Information Index* across metropolitan areas (2014).


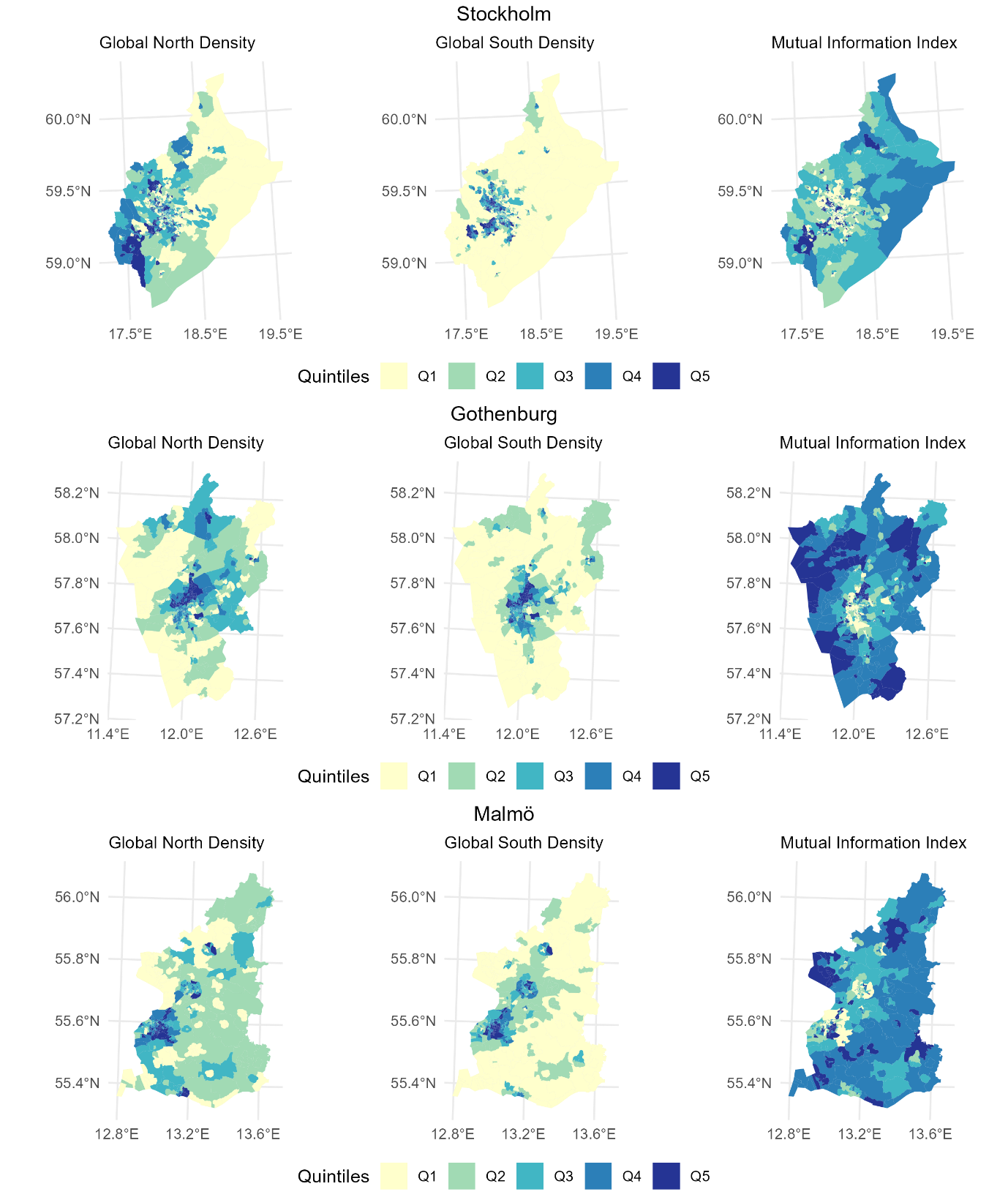


*The three group Mutual Information Index included groups categorized as native-born, foreign-born from the Global North, and foreign-born from the Global South.
