# Supplementary material for: A matter of measurement? A Swedish register-based study of migrant residential segregation and all-cause mortality
Source: SSM Popul Health. 2025 Mar 27;30:101793. doi: 10.1016/j.ssmph.2025.101793 (PMC12005324; doi:10.1016/j.ssmph.2025.101793)
Supplement: Multimedia component 5 [file mmc5.docx]

Supplementary File S2. Correlation matrix of the segregation measures by metropolitan area of residence.

|  | **Stockholm** |  |  |  |
| --- | --- | --- | --- | --- |
|  | Migrant Density | Migrant Isolation | Migrant Exposure | Mutual Information Index |
| Migrant Density | 1.00 | 0.97 | 0.86 | 0.74 |
| Migrant Isolation | 0.97 | 1.00 | 0.96 | 0.56 |
| Migrant Exposure | 0.86 | 0.96 | 1.00 | 0.31 |
| Mutual Information Index | 0.74 | 0.56 | 0.31 | 1.00 |
|  |  |  |  |  |
|  | **Gothenburg** |  |  |  |
|  | Migrant Density | Migrant Isolation | Migrant Exposure | Mutual Information Index |
| Migrant Density | 1.00 | 0.95 | 0.88 | 0.78 |
| Migrant Isolation | 0.95 | 1.00 | 0.98 | 0.56 |
| Migrant Exposure | 0.88 | 0.98 | 1.00 | 0.39 |
| Mutual Information Index | 0.78 | 0.56 | 0.39 | 1.00 |
|  |  |  |  |  |
|  | **Malmö** |  |  |  |
|  | Migrant Density | Migrant Isolation | Migrant Exposure | Mutual Information Index |
| Migrant Density | 1.00 | 0.96 | 0.90 | 0.63 |
| Migrant Isolation | 0.96 | 1.00 | 0.98 | 0.42 |
| Migrant Exposure | 0.90 | 0.98 | 1.00 | 0.23 |
| Mutual Information Index | 0.63 | 0.42 | 0.23 | 1.00 |
